# LncRNA TP73-AS1 Exacerbates the Non-Small-Cell Lung Cancer (NSCLC) Process via Regulating miR-125a-3p-Mediated ACTN4

**DOI:** 10.1155/2022/4098271

**Published:** 2022-09-09

**Authors:** Yueyang Tong, Zhemin Feng, Yaqian Li, Chenxi Yan, Wenbo He, Xueyuan Chen

**Affiliations:** Department of Respiratory and Critical Care Medicine, The Affiliated Hospital of Hangzhou Normal University, Hangzhou 310015, Zhejiang, 126 Wenzhou Road, China

## Abstract

**Background:**

LncRNA TP73-AS1 has been revealed to exert a noteworthy impact on the occurrence and advancement of different cancers. In this study, we explored the function of TP73-AS1 in tumor growth, cell progression as well as the relevant molecular mechanism in non-small-cell lung cancer (NSCLC).

**Methods:**

QRT-PCR was employed to assess the expression of TP73-AS1, miR‐125a-3p, and actinin alpha 4 (ACTN4) in NSCLC cells. The biological effect of TP73-AS1 on NSCLC cells was assessed by cell transfection, CCK8, and transwell experiments. We further predicted the interaction among RNAs (TP73-AS1, miR-125a-3p, and ACTN4) through bioinformatics online tools and verified via luciferase reporter, RNA immunoprecipitation, and qRT-PCR assays. Xenograft models of SPC-A1 cells were conducted to test how TP73-AS1 regulates tumorigenesis. Western blot, as well as the immunohistochemistry (IHC) assays, was utilized to measure the expression levels. Functions of TP73-AS1 in NSCLC progression through the miR-125a-3p/ACTN4 axis were investigated by rescue experiments.

**Results:**

Knockdown of TP73-AS1 suppressed the growth and simultaneously attenuated the migration and invasion ability of NSCLC SPC-A1 and A549 cells. Bioinformatics and molecular mechanism assays demonstrated that TP73-AS1 could bind to miR-125a-3p/ACTN4 and regulate their expression. Moreover, the rescued‐function experiment demonstrated that suppressing miR-125a-3p or elevating ACTN4 turned around the suppression effect of sh-TP73-AS1 on NSCLC progression. TP73-AS1 inhibition could also inhibit the NSCLC tumor growth and correspondingly regulated the expression of miR-125a-3p and ACTN4 in the tumor xenograft model.

**Conclusion:**

The present study indicated that TP73-AS1 affects NSCLC progression through a new competitive RNA (ceRNA) regulatory network of miR-125a-3p/ACTN4, providing an underlying target for NSCLC treatment in the future.

## 1. Introduction

Non-small-cell lung cancer (NSCLC) accounts for 85% of lung cancer (nonsmall cell and small cell types), which is recognized as the main cause of disease and deaths worldwide [1, 2]. To date, due to its relapse tendency and limited treatment options, the overall survival rate remains unsatisfactory [3]. Like other cancers, the development of lung cancer is a complex process, including gene mutations and/or disorders, resulting in unusual cell proliferation, invasion, apoptosis, and other behaviors. In any case, these occasions are not completely caught on and there's an urgent need to recognize demonstrative and prognostic biomarkers and unused helpful targets.

With the improvement of high-throughput sequencing technology, thousands of long noncoding RNA (lncRNA) have come into view. Although there is no protein coding element, lncRNA plays an important role in cellular behaviors via its epigenetic functions [4–8]. LncRNA plays a critical role in the growth, migration, invasion, and apoptosis of tumors, and is included within the event and advancement of tumors [9–11]. To provide patients with better treatment results, researchers have been committed to finding various effective tumor markers for a long time, among which lncRNA has vital potential application esteem within *n* the diagnosis and treatment of threatening tumors.

TP73-AS1 (P73 Antisense RNA 1T) is also named p53-dependent apoptosis modulator (PDAM), and its encoding gene is in 1p36, containing 11773 bases. Accumulating research has demonstrated the closing associating of the TP73-AS1 with different cancer cell biologies, including their occurrence, progress, and therapeutic resistance [12]. It has been reported that TP73-AS1 presented an upregulating profile in NSCLC patients and may serve as an indicator of the clinicopathological characterization and decreasing survival [13]. Therefore, TP73-AS1 may be considered as a marker for diagnosis and prognosis, and also as a target for NSCLC treatment. MicroRNAs (miRNAs) are identified as the noncoding RNA types with 18–25 nucleotides long [14]. LncRNA interacts with miRNAs, leading to downstream mRNA translation repression or degradation, which is a common mechanism of lncRNA regulation of tumors [14]. Accumulating evidence has shown that TP73-AS1 controls a series of miRNAs and thereby mediates the migration of the cancers, including miR-125a-3p. While the role of the LncRNA TP73-AS1/miR-125a-3p axis in lung cancer is unclear and remains to be explored.

In this study, based on *in vitro* and *in vivo* experiments, we demonstrated an elevation of TP73‐AS1 in NSCLC, in correlating with its promotion of the NSCLC cell growth, migration, and tumor growth. Additionally, we predicted and verified the regulatory mechanism of TP73-AS1 to ACTN4 via competitively binding to miR-125a-3p. Further assay demonstrated that TP73‐AS1 exacerbates the NSCLC cell progression underlying the mechanisms via miR-125a-3p/ACTN4 axis.

## 2. Materials and Methods

### 2.1. Cell Cultural Experiment

SPC-A1 and A549 cell lines were purchased from the Chinese Academy of Sciences Cell Bank (CASCB, China). The cell was thawed and maintained a cultural medium. The medium is composed of DMEM basal (HyClone, USA) in addition to 10% FBS (Gibco, Grand Island, NY, USA), 100 U/mL penicillin-streptomycin (Invitrogen, Carlsbad, CA, USA). Cells were cultured in the incubator at 37°C and with 5% CO_2_.

### 2.2. Fluorescence In Situ Hybridization (FISH) Assay

The FISH assay was performed to prove the TP73-AS1 location in NSCLC cells. Briefly, cells were incubated with 2 × 10^4^ cells/well in 24-well plates, added with buffer, and washed with PBS. Afterward, a 2 *μ*L probe kit (GenePharma, China) was mixed with buffer to obtain 200 *μ*L and maintained at 73°C for 5 min to induce denaturation. Then 200 *μ*L probe mixture was added into each well under dark conditions and was kept overnight, then cells were washed with buffer, and added with DAPI for staining for 15 min under dark conditions. Finally, the cells were observed under a light microscope (Nikon, Japan).

### 2.3. Cell Transfection

Specific downregulation short hairpin RNA (TP73-AS1 (sh-TP73-AS1#1/2), miR-125a-3p mimics, miR-125a-3p, and controls) were purchased from GenePharma (Shanghai, China). pcDNA3.1-TP73-AS1 was created by amplifying the full-length lncRNA TP73-AS1 and cloning it into an empty pcDNA3.1(GenePharma) plasmid. 3′UTR sequences of TP73-AS1 and ACTN4 with wild type or mutant binding sites of miR-125a-3p were cloned onto the pmirGLO vector (Invitrogen). Lipofectamine 3000 (Invitrogen) was adopted for transfection based on the manufacturerʼs prerequisites.

### 2.4. Quantitative Real-Time PCR

Whole cell RNA was extracted with TRIzol reagent (Invitrogen, Carlsbad, CA, USA). PrimeScript™ II 1st Strand cDNA Synthesis Kit (Takara, Japan) was used for producing first-strand cDNA. The expression of RNAs was tested with SYBR Premix Ex TaqII (Takara) and ABI 7500 fast real-time PCR System (Applied Biosystems, USA). The level of mRNA was calculated with the 2^−ΔΔCt^ method, and U6 or GAPDH was set as an internal control. Primer sequences are listed below: TP73-AS1: F, 5′-TCTTCTGCCCAGATATGGC-3′; R, 5′-CTGATGTCCACGCTAACCA-3′; miR-125a-3p: F, 5′-TAGGTCCCTGAGACCCTTTAACC-3′; R, 5′-ACGCCAGGCTCCCAAGAAC-3′; ACTN4, F: 5′-AGGTAACT GGAGCGTCTGTG-3′; R: 5′-TCGGTTTCCCCTAATGGCAC-3′; GAPDH: forward primer, 5′-TCAAGATCATCA GCAATGCC-3′, 5′-CGATACCAAAGTTGTCATGGA-3′; U6, forward primer, 5′-CGCAAGGATGACACGCAAAT-3′, reverse primer, 5′-CGGCAATTGCACTGGATACG-3′.

### 2.5. Cell Proliferation Assay

4000 cells/well in 96-well plates for testing cell growth. Cells were incubated for different durations including 0, 24, 48, and 72 h. At the aforementioned time points, we added the Cell Counting Kit-8 (MCE, USA) reagent into each well and maintained it for 4 h. Cell growth value was tested with the absorbance at a wavelength of 450 nm.

### 2.6. Transwell Assay

Cell suspension (1 × 10^5^ cells/well (200 *μ*L/well)) was planted in the above chamber (Millipore, Billerica, MA, USA). A normal medium of 600 uL was covered in the below chamber. Migrated cells to the below chamber were fixed after 48 h of incubation. Then the cells were stained with 0.1% crystal violet (Beyotime, Shanghai, China). The staining cells were counted under microscopy. The above chamber was precoated with matrigel chamber was used for evaluating cell invasion (BD Biosciences, San Jose, CA, USA), and an experimental procedure was conducted as described above.

### 2.7. Identification of Targeted miRNAs and mRNAs

The interaction between TP73-AS1 and miRNAs was predicted with software including StarBase (https://starbase.sysu.edu.cn/) and RNA22 (https://cm.jefferson.edu/rna22/). GSE25508 (included 26 lung cancer and normal samples) and GSE51853 (included 126 lung cancer and 5 normal samples) miRNA expression profiles were collected from the GEO database (https://www.ncbi.nlm.nih.gov/geo/). Online GEO2R (https://www.ncbi.nlm.nih.gov/geo/geo2r/) was used to screen differential miRNA expression with a threshold of |log2 fold-change (FC)|> 0.5 and *P* < 0.05. The intersections of miRNAs among 4 groups (including miRNAs predicted by Starbase and RNA22 as well as miRNAs with abnormally low expression in tumor tissues from GSE25508 and GSE51853 datasets) were analyzed using Venn analysis (https://bioinformatics.psb.ugent.be/webtools/Venn/). The interacting targets of miR-125a-3p were predicted via Targetscan7.2 (https://www.targetscan.org/vert_72/), miRwalk (https://mirwalk.umm.uni-heidelberg.de/), Starbase, and RNA22. The intersections of mRNAs among 4 groups were determined using Venn analysis.

### 2.8. Luciferase Assay

Sites of potential binding in TP73-AS1, ACTN4 (TP73-AS1-WT, ACTN4-WT)), mutation sequence (TP73-AS1-MUT, ACTN4-MUT) were inserted into dual-luciferase reporter vector (pmirGLO, Promega, USA). Plasmids were cotransfected with miR-125a-3p mimic or negative control mimic. The expression level was evaluated by dual-luciferase reporter assay system (Promega, USA).

### 2.9. RNA Immunoprecipitation (RIP) Assay

The RIP assay was processed by following the previous report with IgG control or anti-Ago2 monoclonal antibody (Sigma-Aldrich) [15].

### 2.10. Western Blot

The Western blot was performed by following the previous report [16]. Primary antibodies of ACTN4 (ab108198, Abcam, USA) and GAPDH (AF7021, Affinity Biologicals) was incubated with the membrane at 4°C overnight. After washing on the second day, HRP-conjugated secondary antibodies were incubated with membranes at 37°C for 2 h. Protein bands were shown and analyzed by an ECL detection kit (Thermo, Waltham, MA, USA) with the chemiluminescence system (Bio-Rad, USA).

### 2.11. Xenograft Model

Twelve BALB/c nude mice (5-week-old) were treated with subcutaneous injection with 1 × 10^6^ cells with sh-TP73-AS1 or sh-NC, with six rats in each group. Then measure the tumor volumes every 3 days by calculating length × width^2^ × 0.5. After being sacrificed, xenograft tumors were collected, weighed, and processed with immunohistochemistry (IHC) staining on 31 days.

### 2.12. IHC Analysis

Immunostaining was performed according to the standard protocols by Servicebio (Wuhan, China). The primary antibodies of Ki-67 (AF0198, Affinity Biologicals) and ACTN4 (ab108198, Abcam, USA) at 4°C for 12h. HRP-labeled goat anti-rabbit IgG H&L were then incubated with sections (ab97080, Abcam, USA) at room temperature, and then visualized with diaminobenzidine (DAB, Servicebio) hematoxylin (Servicebio).

### 2.13. Statistical Analysis

All results were displayed as mean ± standard deviation (SD). The data were analyzed and presented with SPSS 16.0 (SPSS Inc., Chicago, IL, USA). Statistical analysis was performed with Student's *t*-test or one-way ANOVA. Statistical significance was set as *P* < 0.05.

## 3. Results

### 3.1. Downregulation of TP73-AS1 Attenuates the Progression of NSCLC

**Figure 1:**
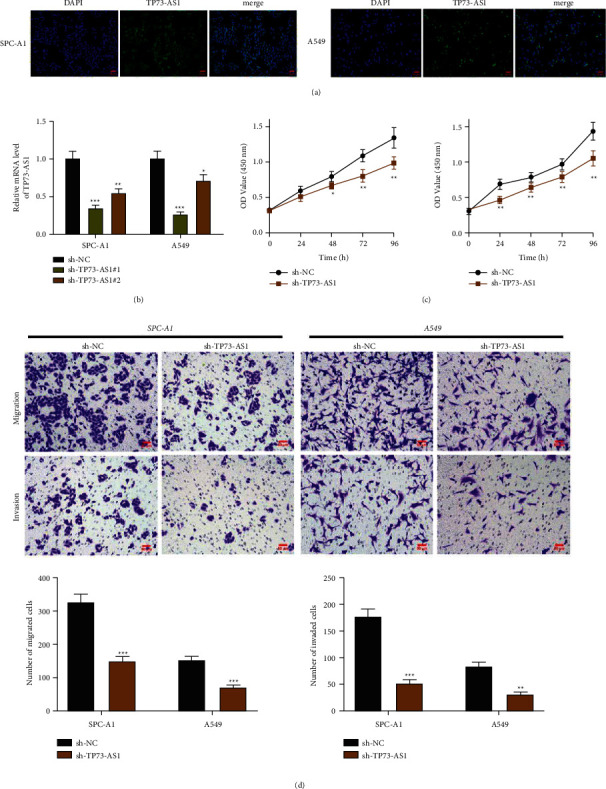
Downregulation of TP73-AS1 inhibits the NSCLC progression. (a) Fluorescence in situ hybridization assay for TP73-AS1 in SPC-A1 and A549 cells. (b) QRT-PCR assay to show the TP73-AS1 knockdown effects in the SPC-A1 cell line and A549 cell line. (c) Proliferation ability assessed by CCK-8 in the SPC-A1 cell line and A549 cell line treated with sh-TP73-AS1 or sh-NC. (d) Transwell picture to represent the migration and invasion of SPC-A1 cell line and A549 cell line after transfected sh-TP73-AS1 or sh-NC (magnification = 400*x*; Scale bar = 50 *µ*m). Compared to the sh-NC group, ^*∗*^*P* < 0.05,  ^*∗∗*^*P* < 0.01,  ^*∗∗∗*^*P* < 0.001.

Firstly, we performed the Fluorescence in situ hybridization assay to confirm that TP73-AS1 is mainly expressed in the cytoplasm, cell nucleus was also found in the expression (Figure 1(a)). Afterward, we used specific shRNAs to knock down (KD) the abundance of TP73-AS1 in SPC-A1 and A549 cells for investigating its roles in the development of NSCLC. qRT-PCR was performed to verify the KD rate (Figure 1(b)). As the sh-TP73-AS1#1 cells had the better KD rate, this group cell was used for the subsequent experiments. In the CCK-8 assay, the growth curves demonstrated that depletion of TP73-AS1 inhibited proliferation of SPC-A1 and A549 in a significant manner (Figure 1(c)). Transwell experiment implied that downregulation of TP73-AS1 resulted in the noteworthy reduction of migrating and invasive cells (Figure 1(d)). Collectively, TP73-AS1 take participated in regulating the growth, migration, and invasion of NSCLC cells.

### 3.2. TP73-AS1 Targets on miR-125a-3p in NSCLC

**Figure 2:**
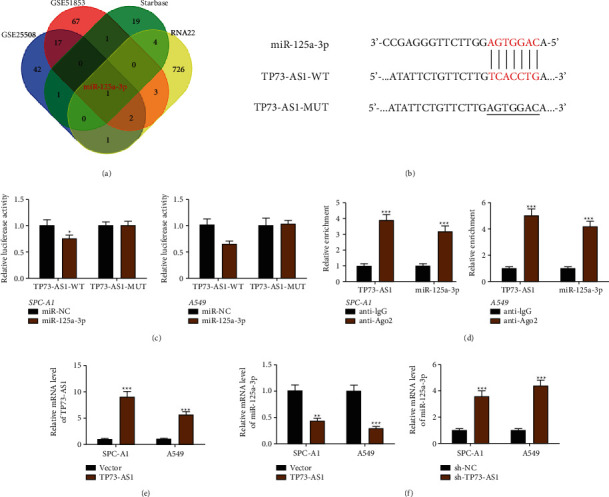
TP73-AS1 targets and negatively regulates miR-125a-3p in NSCLC. (a) Venn diagram of 4 groups (including miRNAs predicted by Starbase and RNA22 as well as miRNAs with abnormally low expression in tumor tissues from GSE25508 and GSE51853 datasets). (b) Potential binding sites between TP73-AS1 and miR-125a-3p. (c) Luciferase reporter showed the interaction between TP73-AS1 and miR-125a-3p. (d) Binding relationship verified by RNA immunoprecipitation assay. (e) qRT-PCR to show the efficiency of TP73-AS1 overexpression. (f) qRT-PCR assay to demonstrate the miR-125a-3p expression after TP73-AS1 overexpression or knockdown. Compared to the relevant miR-NC group, vector group or sh-NC group, ^*∗*^*P* < 0.05,  ^*∗∗*^*P* < 0.01,  ^*∗∗∗*^*P* < 0.001.

The combination of TP73-AS1 targeted miRNAs was predicted from bioinformatics software Starbase, RNA22, as well as miRNAs with abnormally low expression in tumor tissues from GSE25508 and GSE51853 datasets, and were clustered by Venn investigation. The results replied that only one miRNA (has-miR-125a-3p) appeared in the intersection between these four groups (Figure 2(a)). The binding sequences were appeared in Figure 2(b). A dramatically decreased luciferase activity was observed in the cells with cotransfection with TP73-AS1-WT plus miR-125a-3p compared with the miR-NC, while no effect was observed on the mutant form or TP73-AS1 (Figure 2(c)). Therefore, the direct interaction of TP73-AS1 with miR-125a-3p occurs through the putative binding sites. Compared with the anti-IgG control, the RIP assay presented a dramatic increased TP73-AS1 and miR-125a-3p in Ago2-containing miRNAs (Figure 2(d), suggesting TP73-AS1 could be combined with the RISC complex. We also overexpressed TP73-AS1 in SPA-A1 and A549 cells (Figure 2(e)). As illustrated in Figure 2(f), upregulation of TP73-AS1 caused the decreased expression of the miR-125a-3p, while TP73-AS1 depletion showed completely contrary results. These discoveries illustrated that miR-125a-3p could target TP73-AS1 and was contrarily tweaked by TP73-AS1.

### 3.3. ACTN4 Is Targeted with miR-125a-3p and Modulated by TP73-AS1

**Figure 3:**
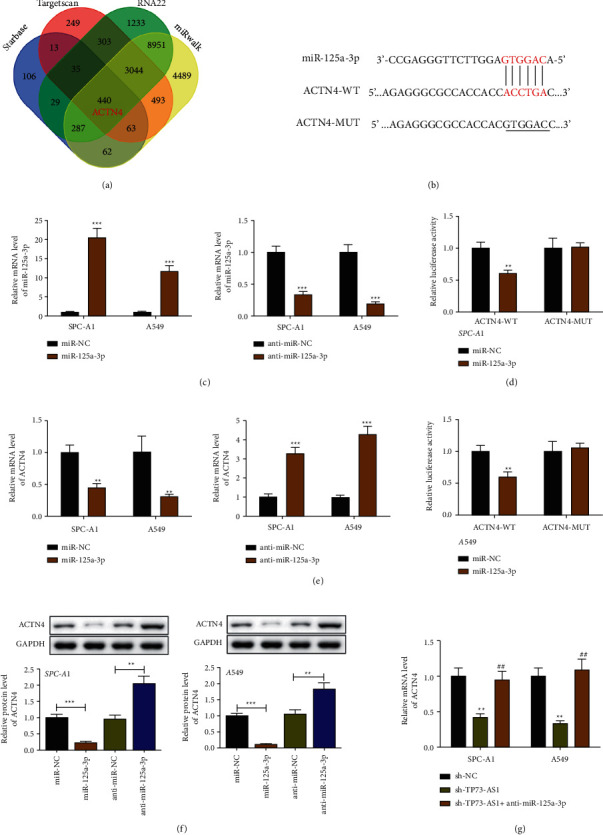
ACTN4 targets on miR-125a-3p via TP73-AS1. (a) Venn diagram to show predicting targets mRNAs of miR-125a-3p. (b) Potential binding sites between miR-125a-3p and ACTN4. (c) QRT-PCR assay to confirm overexpression or knockdown of miR-125a-3p. (d) The luciferase reporter experiment indicates the interaction of miR-125a-3p with ACTN4. (e) MRNA expression of ACTN4 after overexpression or knockdown of miR-125a-3p. (f) Immunoblot bands and statistical analysis to show the protein level of ACTN4. (g) MRNA expression of ACTN4 in SPC-A1 cell line and A549 cell line treated with sh-TP73-AS1 and miR-125a-3p inhibitor. Compared to the relevant miR-NC group, anti-miR-NC group or sh-NC group, ^*∗*^*P* < 0.05,  ^*∗∗*^*P* < 0.01,  ^*∗∗∗*^*P* < 0.001; compared to the sh-TP73-AS1group, ^##^*P* < 0.01.

Subsequently, we discussed the predictable miR-125a-3p targets. 440 mRNAs were screened by the intersection of Venn diagrams of target mRNAs predicted by Targetscan7.2, miRwalk, Starbase, and RNA22 online software, and ACTN4 was focused among them (Figure 3(a)). The binding sequences were displayed in Figure 3(b). QRT-PCR results affirmed that miR-125a-3p significantly increased with its mimics and was sharply silenced when receiving the inhibitor (Figure 3(c)). Luciferase intensity of cells in ACTN4-WT + miR-125a-3p was remarkably lower than ACTN4-WT + miR-NC. No remarkable difference showed between the ACTN4-MUT + miR-125a-3p group and the ACTN4-MUT + miR-NC group (Figure 3(d)). The results suggested that ACTN4 is directly targeted by miR-125a-3p. The consequent investigation appeared that ACTN4 expression was essentially diminished by the cells transfected with miR-125a-3p (Figure 3(e)). Further Western blot assays yielded similar results (Figure 3(f)). Additionally, ACTN4 expression was remarkably suppressed with the knockdown of TP73-AS1 and was recovered due to miR-125-3p silence (Figure 3(g)). Taken together, ACTN4 exerts a direct interaction with miR-125a-3p and is modulated by TP73-AS1.

### 3.4. TP73-AS1 Advances NSCLC Development by Targeting miR-125a-3p/ACTN4 Axis

**Figure 4:**
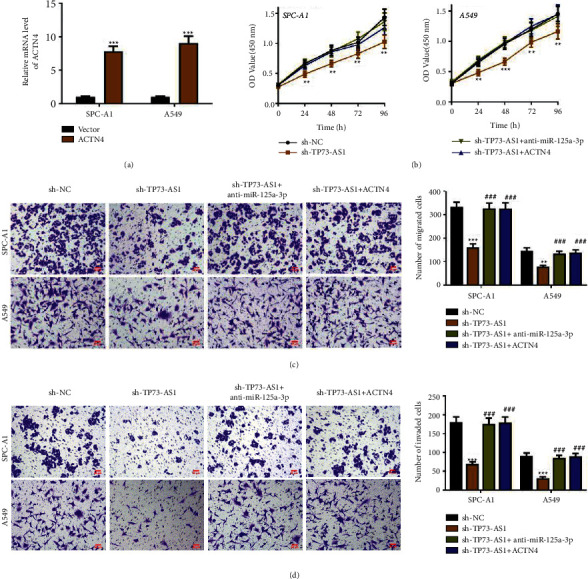
TP73-AS1 promotes NSCLC progression by targeting miR-125a-3p/ACTN4 axis. (a) The qRT-PCR result shows the overexpression efficiency of ACTN4. (b) The proliferation in the CCK-8 test. ((c) and (d)) Cell migration and invasion in transwell test (magnification = 400*x*; scale bar = 50 *µ*m). Compared to the relevant vector group, or sh-NC group, ^*∗*^*P* < 0.05,  ^*∗∗*^*P* < 0.01,  ^*∗∗∗*^*P* < 0.001; compared to the sh-TP73-AS1group, ^###^*P* < 0.001.

We also employed the rescue assays through transfection methods. As revealed in Figure 4(a), ACTN4 was significantly upregulated with the transfection of pcDNA3.1-ACTN4. Cell viability was viably hindered due to TP73-AS1 knockdown, which was protected by miR-125a-3p downregulation or ACTN4 overexpression (Figure 4(b)). Meanwhile, the attenuated cell migration and invasion mediated by TP73-AS1 downregulation were compromised with the restraint of miR-125a-3p and overexpression of ACTN4 (Figures 4(c) and 4(d)). Overall, TP73-AS1 advances NSCLC development by targeting miR-125a-3p/ACTN4 axis.

### 3.5. TP73-AS1 Depletion Inhibits the Xenograft Tumor Growth of NSCLC

**Figure 5:**
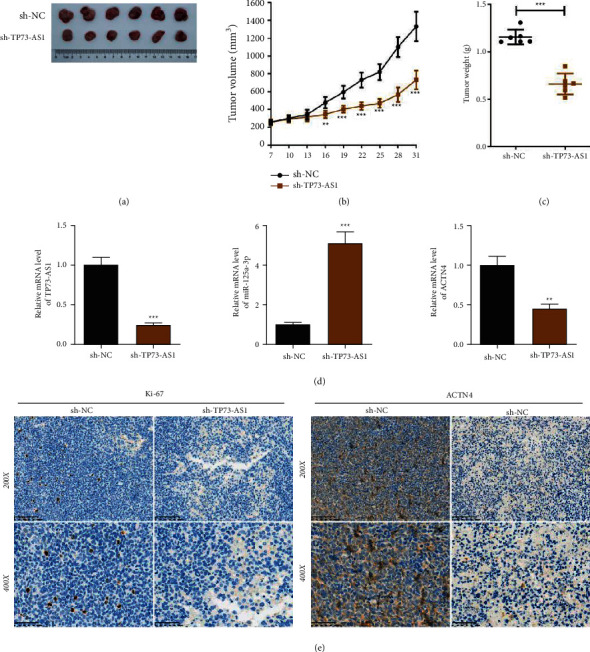
TP73-AS1 depletion inhibits the xenograft tumor growth of NSCLC. (a) Bright field tumor samples to reflect the tumorigenesis of SPC-A1 treated with sh-TP73-AS1. (b) Tumor growth curves of mice after injected with different treated SPC-A1 cells. (c) Statistical graph of tumor weight. (d) The mRNA level of TP73-AS1, miR-125a-3p, and ACTN4 in subcutaneous tumors was measured via qRT-PCR assay. (e) Representative images of Ki67-and ACTN4-positive sections of subcutaneous tumor tissues by IHC assay (top panels: magnification = 200*x*; Scale bar = 100 *µ*m; bottom panels: magnification = 400*x*; scale bar = 50 *µ*m). Compared to the relevant sh-NC group,  ^*∗∗*^*P* < 0.01,  ^*∗∗∗*^*P* < 0.001.

To further test the *in viv*o impact of TP73-AS1/miR-125a-3p/ACTN4 axis, we set up the subcutaneous xenograft tumor model with SPC-A1 treated with sh-TP73-AS1. As indicated, transfecting the sh-TP73-AS1 decreased the volumes and weights of the xenograft tumor (Figures 5(a)–5(c)). This knockdown also decreased TP73-AS1 and ACTN4 and increased the miR-125a-3p level (Figure 5(d)). IHC showed that sh-TP73-AS1 established the decreased ki-67 and ACTN4 expressions (Figure 5(e)). Therefore, depletion of TP73-AS1 inhibits xenograft tumor growth.

## 4. Discussion

LncRNAs usually showed the abnormal expression profile correlating with the progressions of various types of cancer. Emerging evidence supports that those lncRNAs plays critically in regulating tumor occurrence via the transcriptional and post-transcriptional levels. A Previous study indicates that elevating TP73-AS1 in NSCLC tissues could predict the poor patient survival rate [13]. Consistent with the previous studies [15, 17, 18], we found that the depletion of TP73-AS1 restrained the progression of NLSCLS. Therefore, this study implies that TP73-AS1 has a significant tumorigenic effect in the development of NSCLC.

Recent reports have demonstrated that TP73-AS1 could sponges miRNAs, such as miR-141-3p [17], miR-449a [15], and microRNA-34a-5p [18], thereby affecting the biological progress of NSCLC. However, whether there are other miRNAs participating in the regulatory network of TP73-AS1 for controlling the development of NSCLC is still worthy of exploration. Mechanistically, miR-125a-3p was indicated as the target of TP73-AS1 via bioinformatics prediction, and the targeting relationship has been verified through the dual luciferase and RIP assays. In addition, qRT-PCR demonstrated that TP73-AS1 strikingly enhanced miR-125a-3p expression. Hence, the regulatory effect of TP73-AS1 may be acted through its competitive binding to miR-125a-3p.

MiRNAs usually take part in most biological processes through the post-transcriptional modification of gene expression [19, 20]. MiR-125a-3p is highly conserved during the whole evolutionary process [21, 22]. MiR-125a-3p was reduced in NSCLC and it performs as tumor suppressor miRNA to tumor development via regulating the level of target genes [23–25]. ACTN4 belongs to the actin cross-linked protein family and has been revealed to elicit a cancer-promoting effect on the progression of malignancies [26–28]. As early as 1998, Honda et al. first confirmed that ACTN4 is a gene related to breast cancer metastasis, and then found that ACTN4 can accelerate the invasion capacity of colorectal cancer cells and is related to lymph node metastasis [29]. Liu et al. recommended that ACTN4 expression was suppressed in gastric cancer patients with metastasis, and ACTN4 could reduce the adhesion of gastric cancer cells and increase the ability of metastasis and invasion [30]. Jung et al. found that ACTN4 can be involved in regulating the characteristics of tumor stem cells, thus leading to drug resistance to cervical cancer [31]. Moreover, ACTN4 has been reported as a potential biomarker for NSCLC and promotes migration and invasion [32–34]. ACTN4 might exert multiple functions in tumor tissues, which could also be related to the degree of malignancy of other tumors. Herein, targeting ACTN4 may be a new regulatory mechanism for NSCLC. In this study, we found that ACTN4 could target miR-125a-3p in NSCLC cells. More importantly, ACTN4 overexpression could reverse the inhibition effect of sh-TP73-AS1 on NSCLC cell growth and migration, which indicated that ACTN4 is involved in the regulatory network of TP73-AS1/miR-125a-3p/ACTN4 and affecting NSCLC progression.

Noteworthy, the hindrance of miR-125a-3p nullified the effects of sh-TP73-AS1 NSCLC progressing. Our rescue experiments suggest that TP73-AS1 deletion inhibited the progression of NSCLC cells by miR-125a-3p on ACTN4. More importantly, xenograft assays demonstrated that TP73-AS1 KD inhibits tumor growth via modulating the miR-125a-3p/ACTN4 axis, as expanded miR-125a-3p level and diminished ACTN4 level were observed in tumor tissues. Therefore, combined with previous evidence [13, 15, 18, 33], ACTN4 and TP73-AS1 may have similar abnormally high expression in NSCLC tissues. Specifically, the interaction among TP73-AS1, miR-125a-3p as well as ACTN4 may be a critical regulatory pathway to the progression of NSCLC.

In summary, after carrying out a series of biofunctional, molecular, and rescue analyses, our study concluded that suppressing TP73-AS1 obstructs the NSCLC progressing through miR-125a-3p/ACTN4 axis. This finding may offer new insight for early prevention and treatment of NSCLC.

## Data Availability

All data of this study are included in this article.
